# Effect of *Lactiplantibacillus plantarum* and *Saccharomyces cerevisiae* Inoculation on Quality of *Suanyu*: Focusing on the Protein Degradation and Flavor Profiles Analysis

**DOI:** 10.3390/foods15152626

**Published:** 2026-07-27

**Authors:** Qiang Zhang, Yaru Li, Wenrui Liang, Naiyong Xiao

**Affiliations:** College of Food Science and Technology, Guangdong Ocean University, Zhanjiang 524088, China; qzzz1274@163.com (Q.Z.); lyr520@stu.gdou.edu.cn (Y.L.); liangwr@stu.gdou.edu.cn (W.L.)

**Keywords:** *Lactiplantibacillus plantarum*, *Saccharomyces cerevisiae*, fermentation, protein degradation

## Abstract

To investigate the effects of *Lactiplantibacillus plantarum* (LP) and *Saccharomyces cerevisiae* (SC) inoculation on quality of Suanyu, the related chemical parameters such as moisture content, protein degradation index, tricarboxylic acid (TCA) cycle soluble peptides, SDS-PAGE, free amino acids, volatile flavor compounds, and sensory characteristics were analyzed. Natural fermentation (NF), LP fermentation, and SC fermentation were used as control groups. The results indicated that Suanyu samples inoculated with LP and SC (MF) exhibited a significant reduction in moisture content, while simultaneously accelerating the degradation of myofibrils and myosin. This was confirmed by the fading or disappearance of corresponding bands in SDS-PAGE, as well as the appearance of high-molecular-weight protein aggregates. Furthermore, the content of TCA-soluble peptides and free amino acids in the MF group also increased significantly after fermentation, particularly umami amino acids (20.56%) and sweet amino acids (34.14%). With regard to volatile compounds, the addition of SC made a significant contribution to the formation of alcohols and esters. Sensory evaluation confirmed that the MF group exhibited superior performance in terms of flavor, aroma, and overall sensory characteristics. Overall, the combination of LP and SC better promotes protein hydrolysis and the formation of flavor compounds in Suanyu. This study provides a theoretical basis for optimizing starter cultures and promoting the industrial production of high-quality fermented fish products.

## 1. Introduction

Suanyu is one of the traditional fermented fish in China, which is preferred by consumers due to its unique flavor and taste [1]. Suanyu is mainly made from grass carp, carp, and other freshwater fish. Freshwater fish are characterized by high protein and moisture contents; however, due to environmental factors and the rapid action of endogenous enzymes and ambient microorganisms, they are highly susceptible to post-mortem spoilage, thereby limiting their shelf life and commercial value. Therefore, it is necessary to develop suitable processing methods to improve the processing rate and added value of freshwater fish.

Fermentation, as a traditional processing and preservation technique, is widely applied to enhance the distinct flavor profiles and textural attributes of fish products [2], thereby effectively improving the commercial and utilization value of freshwater fish. In addition, the utilization value of freshwater fish is effectively improved. It is a low cost, convenient, and energy saving method for fish preservation. Traditional fermented fish is mostly made from fish and salt, wine lees, cornmeal, and other ingredients mixed, and sealed for a period of time. Because traditional fermentation is spontaneous, the fermentation conditions are difficult to control, which is not conducive to industrial quality assurance and sustainable production. To solve this problem, one or more exogenous starter cultures are added to fermented fish to control the fermentation process, thereby standardizing product quality characteristics [3]. Lactic acid bacteria (LAB) and *Saccharomyces* spp. are used as the main starter for fermenting fish. Due to the selection of specific enzymes and microorganisms, inoculation of microbial fermentation makes the hybrid bacteria effectively limited, the stability and safety of the fermentation process are guaranteed, and the fermentation time can be shortened and the flavor of the product can be improved [4,5]. Studies have reported that, compared with single-culture fermentation, the mixed culture fermentation strains can enhance competitiveness so that the growth of harmful microbes is suppressed, guaranteeing the quality and safety of fermentation products [6,7]. In co-fermentation, while extensive research has focused on the synergistic mechanisms within bacterial systems, such as LAB and LAB [8] or *Staphylococcus* spp. [9], the interactions and co-fermentation dynamics between beneficial bacteria and yeasts in fermented fish products remain less explored.

In recent years, LAB has been widely used in most fish fermentation industries, which can reduce the pH and thus inhibit the proliferation of spoilage microorganisms [10]. Additionally, LAB plays a part in protein degradation. Protein degradation is usually a process of releasing amino acids and peptides caused by the action of endogenous enzymes and microorganisms. It has been reported that *L. plantarum* promotes protein degradation in sausage [11]. Compared with LAB, the research on yeast in the fermentation of meat products is relatively small. However, the addition of yeast can improve the color and taste of fish. Flores et al. found that the addition of *Dabaryomyces hansenii* promoted the sensory properties of meat products [12]. Moreover, the acidification of the sample matrix caused by LAB may favor the growth of yeast, which in turn may release vitamins and other growth factors to stimulate the growth of LAB [13]. It has been proved that LAB combined with yeast fermentation can improve the flavor of meat products [14]. However, there are few reports about the effect of combined fermentation on protein degradation.

Therefore, in order to investigate the effect of the combination of LAB and yeast on the protein degradation characteristics of Suanyu, *L. plantarum* and *S. cerevisiae* were selected as starter cultures, aiming to explain the mechanism through the related indexes of protein degradation. This research may offer some valuable information for the improvement of the fermentation process and provides a theoretical basis for the industrial production of high-quality fermented Suanyu products.

## 2. Materials and Methods

### 2.1. Materials

*L. plantarum* (No. 192567) and *S. cerevisiae* (No. 336054) was obtained from Beijing Beina Chuanglian Biotechnology Research Institute (8026, 8/F, No. 102, Dongsanhuan South Road, Chaoyang District, Beijing, China). The lyophilized strains were subcultured and stored at 4 °C. Single colonies of *L. plantarum* were selected and cultured in De Man, Rogosa, and Sharpe (MRS) Broth (Beijing Luqiao Technology Co., Ltd., Beijing, China) at 37 °C for 24 h. Then, 0.1 mL bacterial suspension was inoculated in 10 mL MRS Broth and cultured in 37 °C for 16 h. Single colonies of *S. cerevisiae* were selected and cultured in Yeast Malt (YM) agar (Beijing Luqiao Technology Co., Ltd., Beijing, China) at 30 °C for 24 h, and 0.1 mL bacterial suspension was inoculated in 10 mL YM agar and cultured in 30 °C for 14 h. After culture, the bacterial solution was centrifuged at 4 °C and 9700 rpm for 10 min, the supernatant was removed, and the bacterial cells were collected, washed twice with an equal volume of a sterile 0.85% (*w*/*v*) sodium chloride solution with a pH adjusted to 7.0, then resuspended in a sterile 0.85% (*w*/*v*) sodium chloride solution with a pH adjusted to 7.0. The final concentration was adjusted to 10^8^~10^9^ cfu/mL, corresponding to an OD600 value of 0.813 for *L. plantarum* and 1.118 for *S. cerevisiae*.

### 2.2. Sampling

Fifteen live grass carp were purchased at Lingang Agro-Industrial Supermarket in Pudong New Area, Shanghai, China. The live grass carp was stunned, decapitated, the viscera removed, and the back meat was rinsed and cut into rectangular pieces (3 cm × 3 cm × 2 cm). The sample was processed by the method reported in Xu [15]. The back meat, white sugar (2%, *w*/*w*), and salt (3%, *w*/*w*) were mixed, and the mixture was evenly stirred and pickled at 4 °C for 48 h. The marinated fish, the roasted cornmeal (25%, *w*/*w*), and the bacterial solution (1%, *w*/*w*) were mixed thoroughly. The mixture was loaded into a fermenter, sealed with water, and fermented in an incubator at 30 °C for 15 days. Samples were taken at 0, 5, 10, and 15 days for further testing and stored at −80 °C. A total of three groups of fermented samples were designed: NF group (natural fermentation without bacteria), SC group (*S. cerevisiae* inoculated), LP group (*L. plantarum* inoculated), and MF group (*L. plantarum*, *S. cerevisiae* inoculated (1:1 by volume, 1% inoculation)).

### 2.3. Moisture Content and Total Nitrogen (TN)

Determination of moisture contents was performed by the Standard method 930.15 of AOAC [16],TN was determined using the FOSS 8400 Kjeldahl nitrogen analyzer (Kjeltec 8400, FOSS Analytical A/S, Hillerød, Denmark).

### 2.4. Non-Protein Nitrogen (NPN)

NPN was determined according to Chen’s method with a slight modification [17]. The sample (2 g) was taken to be homogenized for 2 min in 10 mL trichloroacetic acid (10%, *v*/*v*). After standing for 2 h at 4 °C and centrifuged at 8000 rpm for 15 min at 4 °C, supernatant (2 mL) was taken and adjusted to 10 mL with distilled water. Then NPN was determined by the FOSS 8400 Kjeldahl nitrogen analyzer.

### 2.5. Protein Degradation Index (PI)

The PI was presented by the ratio of NPN content to TN content [17]. 

### 2.6. Trichloroacetic Acid (TCA)-Soluble Peptidest

TCA-soluble peptides were measured referring to Chu’s method [18] with some modifications. Samples were mixed with 18 mL trichloroacetic acid (5%, *w*/*w*), homogenized for 30 s, and left for 1 h at 4 °C. After standing, centrifuged for 15 min at 4 °C and 10,000 rpm, and filtered. The results were determined by the biuret method and expressed as μmol/g.

### 2.7. Protein Fractionation

The protein fractions in Suanyu sample were separated according to different solubility. Chaves-Lopez’s method [19] was used to determine protein fractionation. Briefly, 2 g sample was homogenized with twenty mL of low-salt buffer (20 mM, pH 6.5), incubated for 15 min under refrigeration, and centrifuged at 10,000 rpm and 4 °C for 15 min to collect the supernatant. This extraction process was repeated twice on the resulting precipitate, and all three supernatants were combined to yield the sarcoplasmic protein fraction. Subsequently, the remaining precipitate was mixed with twenty mL of high-salt buffer (50 mM, pH 6.5), homogenized, allowed to stand undisturbed for 2 h, and centrifuged under the same conditions to obtain the myofibrillar protein fraction. The remaining residue was then treated with ten mL of 5% SDS solution, heated in a water bath at 85 °C for 1 h, and cooled with cold water, with the final insoluble precipitate constituting the stroma protein fraction. Finally, the protein content of each isolated fraction was determined quantitatively using the biuret method. The low-salt buffer is “20 mM phosphate buffer (containing 0.05 M NaCl, pH 6.5)” and the high-salt buffer is “50 mM phosphate buffer (containing 0.6 M NaCl, pH 6.5)”.

### 2.8. SDS-PAGE

Sodium dodecyl sulfate polyacrylamide gel electrophoresis (SDS-PAGE) was performed according to Laemmli’s method [20]. A ten percent resolving bisacrylamide and a 5% stacking bisacrylamide were prepared for standard SDS-PAGE analysis, and the sample solution was electrophoreted at 100–120 V until the front marker reached the bottom of the gel. After electrophoresis, the gel was stained with Coomassie brilliant Blue R-250 pairs, then destained with 40% methanol and 10% acetic acid. The Precision Plus Protein Dual Xtra Standard (BioRad, Hercules, CA, USA), from 2 to 250 kDa, was used as the standard.

### 2.9. Free Amino Acids (FAAs)

FAAs in fermented samples were determined by the method of Shi [21] with some modifications. A 2 g sample was mixed with 15 mL of cold trichloroacetic acid (TCA) solution (5%) and homogenized using an FM-200 homogenizer (Shanghai Fokker Equipment Co., Ltd., Shanghai, China) for 1 min. To ensure complete protein precipitation, the homogenate was left undisturbed for 2 h, followed by centrifugation at 10,000 rpm for 15 min at 4 °C and subsequent filtration. Thereafter, a 5 mL aliquot of the supernatant was collected, adjusted to pH 2.0 using 3 M NaOH, and rapidly diluted to a final volume of 10 mL with ultrapure water. Finally, the resulting solution was passed through a 0.22 μm filter membrane and analyzed using an automatic amino acid analyzer (L-8800; Hitachi, Tokyo, Japan).

### 2.10. Volatile Compounds

Volatile compounds in Suanyu samples (5 g) were analyzed via head-space solid-phase microextraction (HS-SPME) coupled with GC-MS, following the method described by Xiao [22] with minor modifications. The sample was placed into a 20 mL sealed headspace vial and equilibrated with the internal standard (2,4,6-trimethylpyridine) at 60 °C for 10 min. The volatile organic compounds were extracted using a Carboxen/Polydimethylsiloxane (CAR/PDMS) fiber (50 μm, 1 cm) for 40 min, and subsequently desorbed in the GC injector at 250 °C for 5 min in split mode. Chromatographic separation was achieved on a DB-5MS capillary column (60 m × 0.32 mm, 1 μm film thickness) with helium as the carrier gas maintained at a constant flow rate of 1.2 mL/min. The oven temperature profile was programmed as follows: initially held at 40 °C for 1 min, ramped to 100 °C at a rate of 5 °C/min, increased to 180 °C at 3 °C/min, and finally raised to 240 °C at 5 °C/min with a final hold time of 5 min. Mass spectrometry was operated in the electron ionization (EI) mode at 70 eV, with the ion source and quadrupole temperatures set at 230 °C and 150 °C, respectively, covering a mass scan range of *m*/*z* 35–450. Volatile compounds were identified by comparing their mass spectra against the Wiley/NIST 2008 library and verifying their linear retention indices (LRIs), and further quantified using the internal standard method based on the peak area ratios.

### 2.11. Sensory Assessment

Ten trained assessors carried out a quantitative descriptive analysis (QDA) of the fermented Suanyu samples in accordance with ISO standards 8586:2023 [23]. The characteristics of the fermented fish shown in Table 1 were assessed (over-acceptance, texture, gloss, odor, and taste), and the scoring standards are shown in the table. The specific standard referred to Yang’s method [24]. Irritating foods were not allowed and mouthwashes with warm water were required before sensory evaluation. Each indicator is scored between 0 and 10; the higher the score, the more likely the sensory indicators representing the sample are to be accepted by consumers.

### 2.12. Statistical Analysis

All experimental assays were carried out in triplicate, with the resulting data presented as the mean ± standard deviation (SD). Statistical significance was assessed using one-way analysis of variance (ANOVA) followed by Duncan’s post-hoc multiple range test, utilizing SPSS software (version 23.0; IBM Corp., Armonk, NY, USA). Differences were considered statistically significant at *p* < 0.05.

## 3. Results and Discussion

### 3.1. Moisture Content Analysis

**Figure 1:**
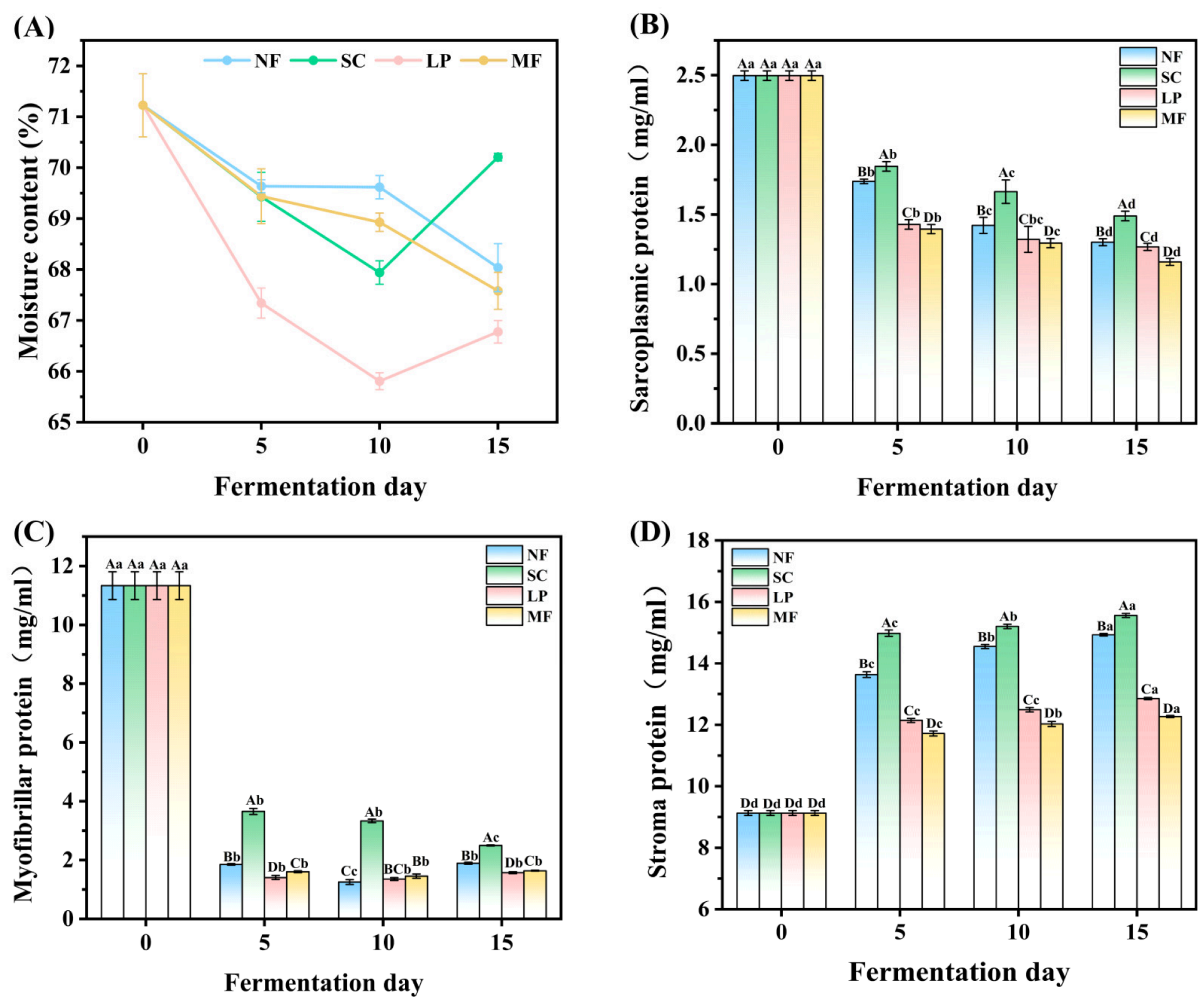
Changes in the fermentation process of Suanyu under different inoculation methods: moisture content (**A**) and protein composition (**B**–**D**). Uppercase letters indicate significant differences between different treatment groups on the same day (*p* < 0.05); lowercase letters indicate significant differences within the same group across different fermentation times (*p* < 0.05).

The changes of moisture content in samples of NF, SC, LP, and MF group during fermentation are shown in Figure 1. As can be seen from Figure 1, at the end of fermentation, the moisture content of all groups ranged from 66.78% ± 0.22% to 70.21% ± 0.07%, which decreased by 1.43% to 6.24% compared with 71.23% ± 0.62% in the unfermented condition. With the progress of fermentation, the moisture content of the NF and MF group decreased gradually. At the end of fermentation, the moisture content of LP group and MF group was significantly lower than that of the natural fermentation group, and the moisture content of LP group was always at a significant minimum during the whole fermentation process. This suggests that the moisture content in fermented Suanyu was effectively reduced by *L. plantarum*. Studies have shown that the decrease of pH after LAB fermentation promotes the activity of endogenous protease, thus promoting the hydrolysis of protein, increasing the content of water-soluble protein, resulting in the decrease of product water retention and water content [25,26].

### 3.2. TN, NPN, and PI Analysis

The changes in TN, NPN, and PI were used to characterize the degradation of protein during fish fermentation. Proteins were extensively degraded into peptides and NPN under the action of various proteases. PI can intuitively reflect the relationship between protein degradation and taste. The changes of TN, NPN, and PI contents in samples of each group during the fermentation process of Suanyu are shown in Table 2. As can be seen from the table, there was no obvious change of trend or difference in TN content of each group during fermentation, which indicated that the addition of *L. plantarum* and *S. cerevisiae* had no significant effect on the change in TN content of fermented fish. Instead, the contents of NPN and PI in the three inoculation groups were significantly higher than those in the NF group, indicating that inoculation with microbial fermentation can promote protein degradation more effectively and produce free amino acids and peptides [22]. At the end of fermentation, the PI value of the MF group and LP group increased to 13.79% ± 0.13% and 12.69% ± 0.20%, respectively. Notably, the MF group exhibited the highest PI value, suggesting a strong synergistic effect between *S. cerevisiae* and *L. plantarum*, where yeast autolysis provides essential nutrients that stimulate bacterial growth and proteolytic activity.

### 3.3. Protein Composition Analysis

The changes in protein composition in the fermentation process of Suanyu are shown in Figure 1B–D. At the beginning of fermentation, myofibrillar protein is the main protein. With the progress of fermentation, the sarcoplasmic protein and myofibrillar protein decreased gradually, while the stroma protein (salt insoluble protein) increased. Similar results were found by Wang et al. during the fermentation of traditional sour fish [27]. This could be due to the denaturation of proteins caused by the decrease in the pH of Suanyu during fermentation, causing them to aggregate and thus become insoluble. At the end of fermentation, myofibrillar protein in the MF group was reduced by 83.30%, which was the highest in all groups.

### 3.4. SDS-PAGE Analysis 

**Figure 2:**
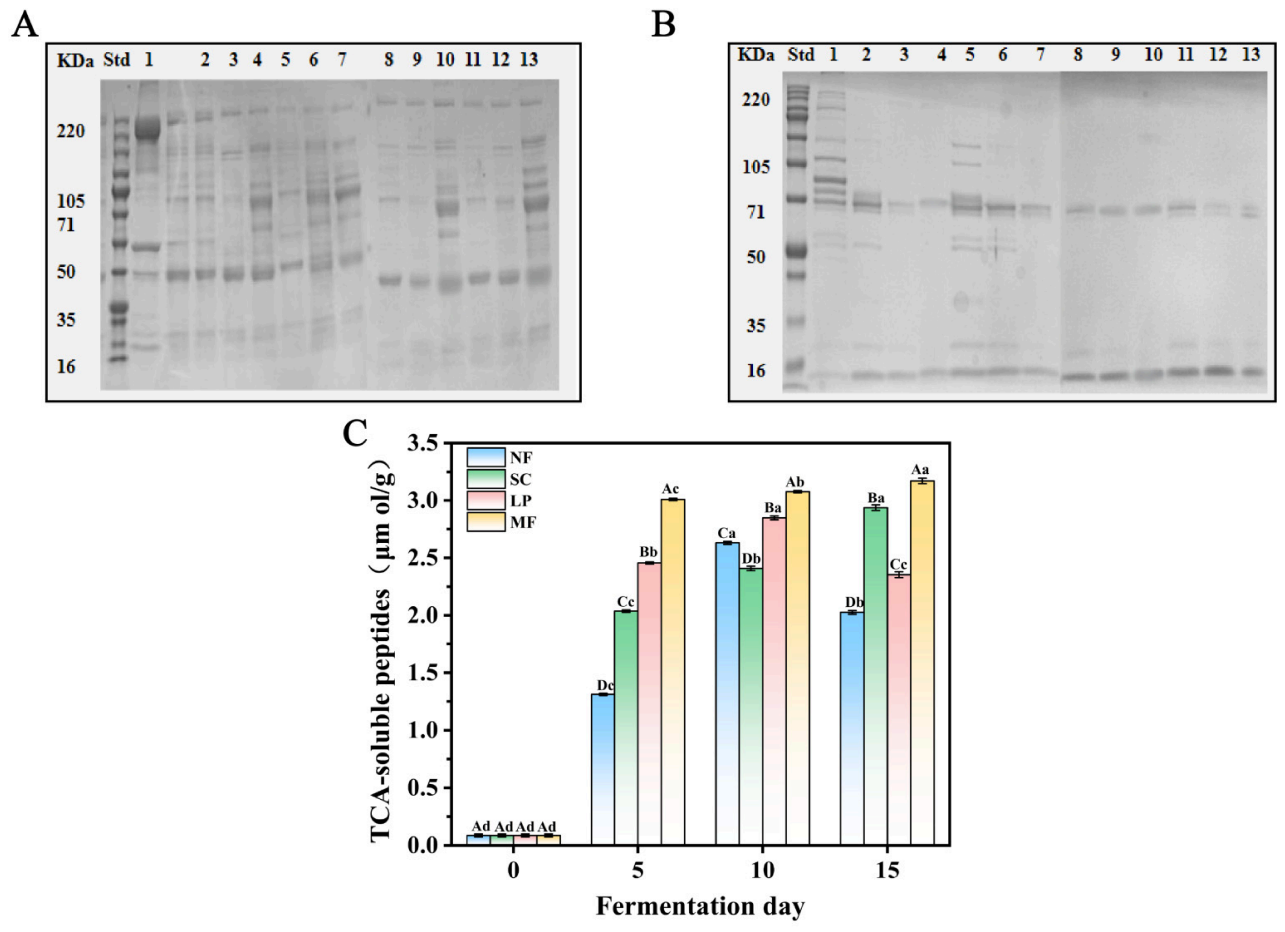
Changes during the fermentation process of Suanyu under different inoculation methods: SDS-PAGE (**A**,**B**), TCA-soluble peptides (**C**). Note: Figure (**A**) shows an SDS-PAGE gel of myofibrillar proteins. Uppercase letters indicate significant differences between different treatment groups on the same day (*p* < 0.05); lowercase letters indicate significant differences within the same group across different fermentation times (*p* < 0.05). Std is the marker; Lane 1 contains the unfermented sample; Lanes 2, 3, and 4 are the NF group at 5 days, 10 days, and 15 days; lanes 5, 6, and 7 are the SC group at 5 days, 10 days, and 15 days; lanes 8, 9, and 10 are the LP group at 5 days, 10 days, and 15 days; lanes 11, 12, and 13 are the MF group at 5 days, 10 days, and 15 days. Figure (**B**) shows an SDS-PAGE gel of sarcoplasmic proteins. Lane Std is the marker; lanes 1, 2, and 3 are for the NF group (5 days, 10 days, 15 days); lanes 4, 5, and 6 are for the SC group (5 days, 10 days, 15 days); lanes 7, 8, and 9 are for the LP group (5 days, 10 days, 15 days); lanes 10, 11, and 12 are for the MF group (5 days, 10 days, 15 days).

The electrophoretic changes of myofibrillar protein and sarcoplasmic protein myofibrillar protein during the fermentation of Suanyu are shown in Figure 2A,B. As can be seen from Figure 2B, the band intensity of these proteins significantly decreased or even disappeared during the whole fermentation process due to protein degradation and acid-induced denaturation. After being inoculated with starter culture, the proteolysis rate of Suanyu was significantly accelerated, and similar changes were found by Fadda et al. in sausage fermented by LAB [11]. Significant degradation of sarcoplasmic protein was observed in all four groups. The band strength decreased significantly at 50–105 kDa, and the protein component was stable at 16 kDa. The protein hydrolysis rate of Suanyu in the MF group and SC group was faster. As shown in Figure 2A, during the whole fermentation process, the changes of myofibrillar protein profiles of the four groups were the same, but the proteolysis of Suanyu samples in the MF group was more intense. The 200 kDa (myosin) and 45 kDa (actin) bands were significantly reduced, and multiple bands appeared simultaneously in the 70–150 kDa range. Similar results were found by Riebroya et al. in fish meal fermented by LAB [28]. SDS-PAGE showed that sarcoplasmic protein and myofibrillar protein of fermented Suanyu could be effectively affected by the inoculation of these microorganisms. However, the effect of *S. cerevisiae* was more obvious, while *L. plantarum* had less effect on protein hydrolysis of fermented Suanyu.

### 3.5. TCA-Soluble Peptides Analysis

The changes of TCA-soluble peptide content in the fermentation process of Suanyu are shown in Figure 2C. The contents of TCA-soluble peptide in each group showed an increasing trend. As reported by Xu et al. in fermented fish sausages, the progressive accumulation of TCA-soluble peptides directly reflects the extent of macromolecular muscle protein hydrolysis driven by enzymatic activities [29]. After pH reduction, proteolysis is promoted by endogenic enzymes and microbial exogenous enzymes in muscle, which produce a large number of peptides and FAA. This was also confirmed by the analysis of protein composition. The contents of TCA-soluble peptide in SC and MF group were significantly higher than that in NF group, suggesting that the addition of *S. cerevisiae* could effectively improve the degree of protein degradation. However, the accumulation of TCA-soluble peptides in the LP group was significantly lower than that in the SC and MF groups. This is highly consistent with the findings of Yang et al., who demonstrated that *L. plantarum* exhibits relatively weak extracellular proteolytic enzyme activity compared to endogenous enzymes in fish matrices, thereby leading to a slower release of low-molecular-weight peptides [30].

### 3.6. FAA Analysis

Free amino acids (FAAs) serve as crucial non-volatile flavor precursors and flavor components that determine the sensory quality of fermented fish products [31]. In this study, compared with single-strain fermentation and natural fermentation, inoculation with a mixed culture of *L. plantarum* and *S. cerevisiae* exerted a significant regulatory effect on protein hydrolysis. 

**Figure 3:**
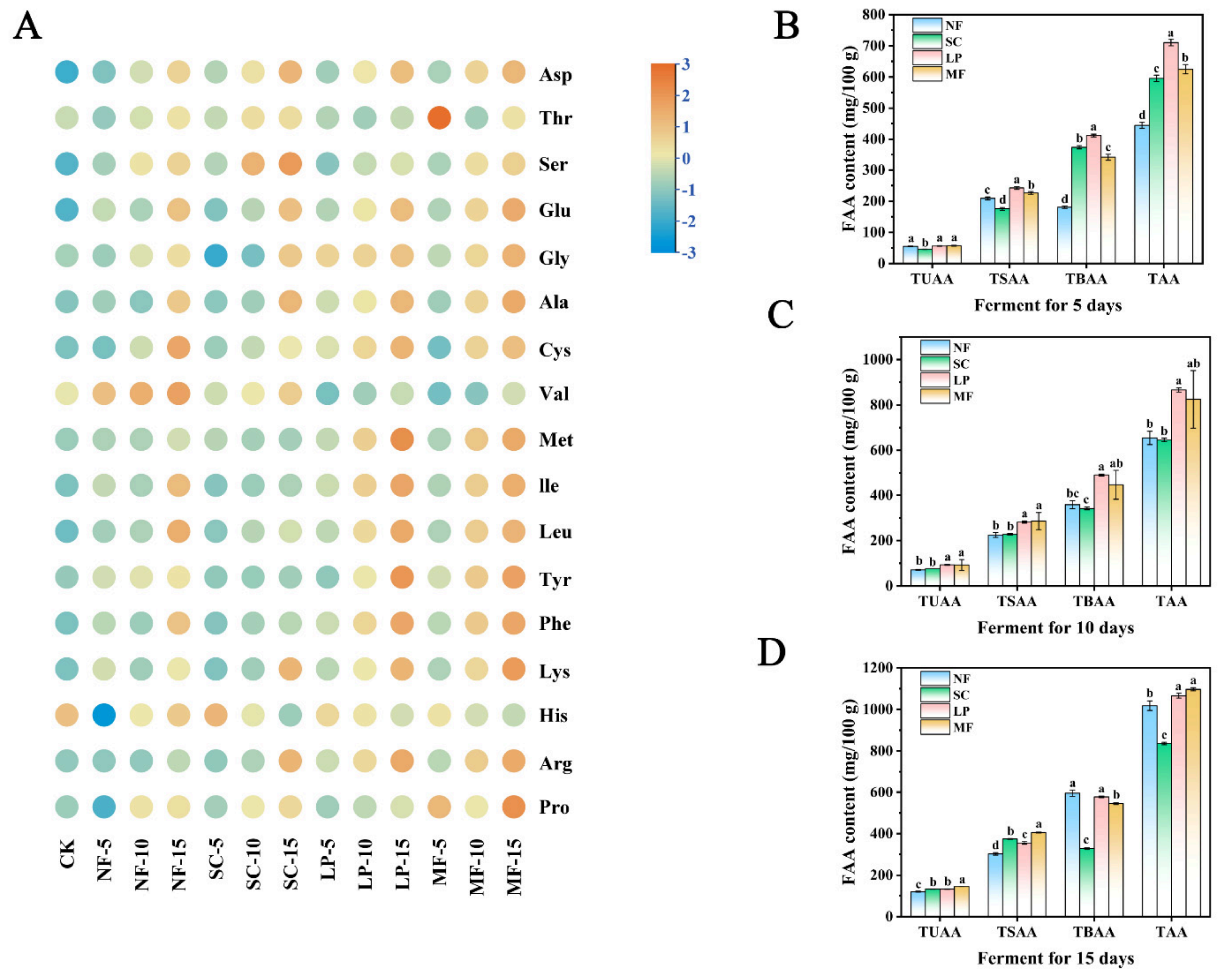
The Changes of FAAs during the fermentation process of Suanyu under different inoculation methods: Amino Acid Distribution Heatmap (**A**), Total amino acid content at 5, 10, and 15 days (**B**–**D**). Note: TAA, total free amino acid; TBAA, total bitter free amino acid; TSAA, total sweet free amino acid; TUAA, total umami free amino acid. Lowercase letters indicate significant differences (*p* < 0.05) in the free amino acid composition between different fermentation samples taken at the same time.

The changes in amino acid content in Suanyu samples prepared using different fermentation methods at various stages of fermentation are shown in Figure 3 and Table S1. As fermentation time increased, the free amino acid content in all groups increased significantly, particularly between days 5 and 15 of fermentation. During the early fermentation stage (days 0–5), threonine levels in the single-strain groups (SC, LP) and the natural fermentation group (NF) remained stagnant or even decreased, whereas the MF group exhibited a significant upward trend, jumping directly from 7.26 ± 0.31 to 33.78 ± 0.66 mg/100 g. This phenomenon indicates that the dual-strain inoculation significantly accelerated the processes of protein hydrolysis and flavor transformation during the late fermentation stage. Glutamic acid and alanine, which contribute to umami flavor, showed the highest increase in the LP group between days 5 and 10 (glutamic acid increased by 20.15 mg/100 g, and alanine by 8.97 mg/100 g). However, during the subsequent final maturation stage (days 10–15), the increase in glutamic acid in the MF group (24.05 mg/100 g) was slightly higher than that in the LP group (23.51 mg/100 g), while alanine also increased by 17.71 mg/100 g. This phenomenon suggests that *L. plantarum* dominated the initial protein hydrolysis during the middle stage, while autolysis or metabolic secretions from *S. cerevisiae* in the later stage may have further promoted the release of umami/sweet flavor components [32]. Although essential branched-chain amino acids such as isoleucine and leucine increased significantly in the MF group, their concentrations were strictly controlled within ranges matching or falling below their high taste thresholds. This means that the bitterness typically associated with deep protein hydrolysis was effectively masked or balanced. Cysteine is highly prone to causing unpleasant rotten egg smell and bitter flavor defects in seafood products. In the NF, SC, and LP groups, cysteine levels showed a significant upward trend by day 15 (reaching as high as 106.19 ± 2.77 mg/100 g in the NF group). However, the MF group successfully maintained cysteine levels at 4.60 ± 0.17 mg/100 g throughout the entire fermentation process. In summary, this study enhanced the activity of endogenous proteases and microbial enzymes by inoculating LP and SC, thereby promoting protein degradation and accelerating fermentation maturation. Concurrently, the competitiveness of proteolytic microorganisms was enhanced, the formation of bitterness was suppressed, and the development of umami and sweetness was promoted. Feng et al. found that the addition of *L. plantarum* and *S. cerevisiae* can promote the production of free amino acids and further improve the flavor of sausages [33], which is consistent with the results of this study.

### 3.7. Volatile Compounds

**Figure 4:**
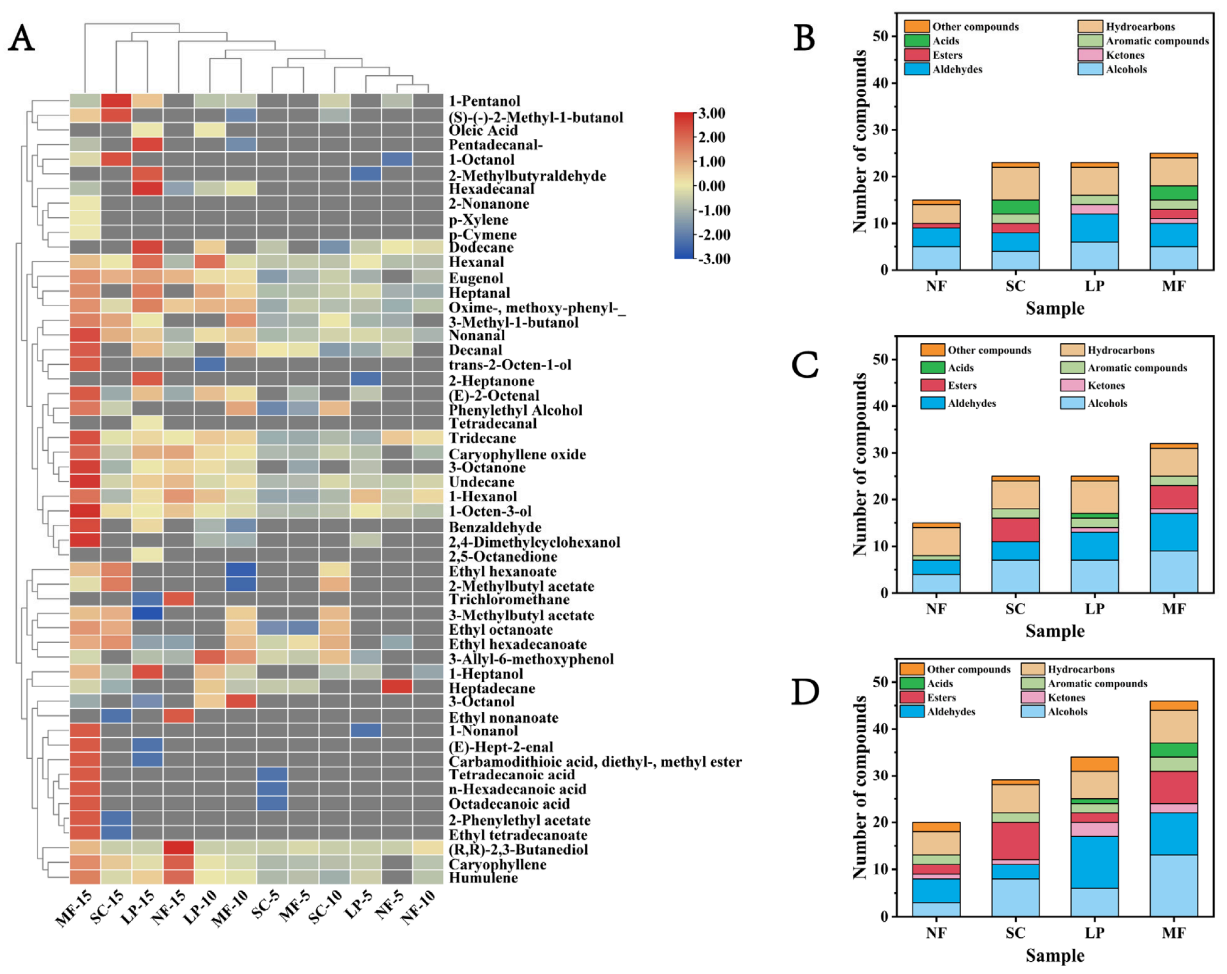
Changes in volatile compounds during the fermentation of Suanyu under different inoculation methods: distribution heatmap (**A**), and compound profiles on days 5, 10, and 15 (**B**–**D**).

Throughout the fermentation process, 54 volatile compounds were detected in all Suanyu samples, including 13 alcohols, 11 aldehydes, four ketones, eight esters, three aromatic compounds, four acids, eight hydrocarbons, and three other compounds. The types and quantities of compounds detected at different fermentation stages in samples treated with different fermentation methods are shown in Figure 4. Alcohols, aldehydes, and esters were the primary volatile compounds throughout the fermentation process. The MF group yielded significantly more volatile compounds and higher concentrations than the other three groups, indicating that the combined inoculation of *L. plantarum* and *S. cerevisiae* confers certain advantages for the production of volatile compounds during the Suanyu fermentation process.

Alcohols are the most abundant class of volatile compounds detected in the MF group and play a significant role in the overall aroma of Suanyu. Through the Ehrlich pathway, yeast can efficiently transaminate, decarboxylate, and reduce free amino acids, ultimately converting them into their respective fusel alcohols [34]. The SC group exhibited a rapid increase in alcohol levels during the mid-fermentation stage (5–10 days), whereas the MF group showed a consistent upward trend in alcohol levels throughout the entire fermentation process. Among these, phenethyl alcohol, characterized by a sweet, rose-like floral aroma, exhibited the most pronounced signal intensity. Aliphatic alcohols derived from fat degradation, such as 1-octen-3-ol (produced from unsaturated fatty acids), which has a mushroom-like aroma, as well as n-hexanol, n-octanol, and 3-methyl-1-butanol, also exhibited significantly higher signal intensities in the MF group compared to the uninoculated group.

Aldehydes are typically produced by the oxidative degradation of unsaturated fatty acids, their low flavor thresholds can significantly affect the overall flavor of seafood products [35]. During the early to mid-fermentation stages (5–10 days), the LP group exhibited higher levels of hexanal (grass-like and fishy odor) and heptanal (citrus aroma), which is consistent with the strong lipolytic activity of *L. plantarum* in the early stages. Crucially, as fermentation progressed into the late stage (10–15 days), the content s of these linear aldehydes, which impart a pungent, raw fishy odor, were significantly lower in the MF group than in the LP group. In contrast, nonanal, which exhibits floral and grassy aromas, became the dominant aldehyde component in the MF group by day 15 of fermentation. The content of benzaldehyde, which imparts mushroom and almond flavors, increased with fermentation time only in the LP and MF groups throughout the process, indicating that this compound is a specific product of *L. plantarum* catabolism. Furthermore, some volatile compounds such as trans-2-octenal, trans-2-heptenal, and hexadecanal, which were not detected in the NF group, also increased significantly in the MF group. Changes in these aldehydes play a crucial role in the formation of the unique flavor of Suanyu sample during the fermentation processes.

Ketones are key volatile compounds that impart the delicate fatty, cheesy, and earthy flavors characteristic of fermented fish. Among the four ketone compounds detected in this study, 3-octonone was identified as the primary flavor-contributing ketone during the fermentation process due to its extremely low detection threshold and its complex sensory profile, which combines mushroom, cheese, and moldy aromas. As fermentation time increased, the 3-octanone content in both the SC and MF groups showed an upward trend, indicating that the addition of *S. cerevisiae* contributes to the enhancement of ketone compounds of Suanyu sample during the fermentation processes.

Esters are extremely important aromatic compounds in fermented fish products, imparting sweet fruity and vinous aromas to the meat products [36]. Throughout the fermentation process, only two and three ester compounds were detected in the NF and LP groups, respectively. In contrast, the SC and MF groups contained 10 and 9 distinct ester compounds, respectively, the vast majority of which were ethyl esters. These ethyl esters (such as ethyl caproate, which has a strong fruity and vinous aroma, and ethyl octanoate, which has a brandy-like aroma) were present in low concentrations during the first 5 days of fermentation, but their levels increased rapidly in the MF group during the later stages of fermentation (10–15 days). This suggests that wine yeast requires a specified duration to accumulate sufficient ethanol through glycolysis, then these alcohols can undergo condensation with the free fatty acids and organic acids continuously hydrolyzed by *L. plantarum*.

Aromatic compounds are hydroxyl-containing derivatives of aromatic hydrocarbons. In this study, two phenolic compounds were detected in all samples, namely eugenol and 3-allyl-6-methoxyphenol. Eugenol is the flavor compound present in the highest concentration during fermentation, imparting a pleasant, clove-like aroma [37]. In the NF group, the content of eugenol decreased as fermentation progressed. However, in the MF group, it showed a steady upward trend from day 5 to day 15, playing a crucial role in the development of the fermentation flavor. The hydrocarbons detected in this study were classified into two categories: alkanes and terpenes. Due to their high threshold, alkanes are considered to contribute very little to flavor [38]. In the MF group, the content of the caryophyllene and humulene increased with fermentation time, imparting a distinctive clove-like flavor to the product. Finally, four long-chain fatty acids, tetradecanoic acid, n-Hexadecanoic acid, octadecanoic acid and oleic acid, were detected during the fermentation process. As the sensory threshold for long-chain fatty acids is relatively high, their direct contribution to the odor is negligible; their primary role is to participate in the formation of esters during the later stages of fermentation [39].

### 3.8. Sensory Assessments

**Figure 5:**
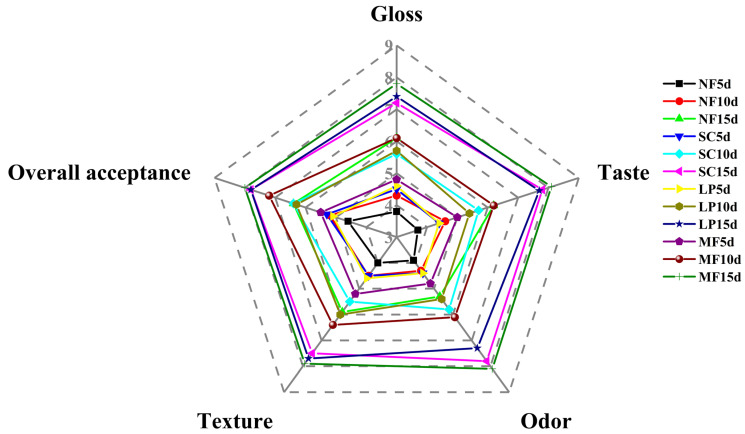
Sensory characteristics of each group of samples during fermentation based on QDA testing.

The sensory scores of all Suanyu samples during the fermentation processes are shown in Figure 5. It can be seen that the sensory scores of the four groups of samples increased with the progress of fermentation, which indicates that fermentation is conducive to improving the edible quality of Suanyu. In addition, the scores of sensory quality in the MF group were significantly higher than those in the other groups. These results indicate that the combined fermentation of *L. plantarum* with *S. cerevisiae* can improve the sensory quality of Suanyu more than that of SC and LP groups with single inoculation. At the end of fermentation, it is worth noting that among the five sensory indexes of the MF group, the scores of taste and odor were the highest, which indicated that inoculation with *L. plantarum* and *S. cerevisiae* could greatly improve the taste and odor of fermented Suanyu products. This may be due to the accumulation of free amino acids and peptides caused by protein degradation, including some sweet amino acids and umami amino acids, so the edible quality of fermented Suanyu was improved, which was consistent with the FAA results.

## 4. Conclusions

The changes of protein degradation and flavor compounds during the fermentation of Suanyu by *L. plantarum* combined with *S. cerevisiae* were studied. With the progress of fermentation, the moisture content of the samples in the mixed fermentation group decreased continuously. The contents of myofibrillar protein and sarcoplasmic protein decreased with the increase of insoluble components during fermentation. SDS-PAGE showed that bands of sarcoplasmic protein and myofibrillar protein became lighter or disappeared with protein degradation, while bands of insoluble protein gradually appeared. Especially, the hydrolysis process of Suanyu inoculated with *L. plantarum* and *S. cerevisiae* was more intense. Compared with *L. plantarum*, the addition of *S. cerevisiae* promoted protein hydrolysis, TCA soluble peptides, and free amino acids, especially umami amino acids and sweet amino acids. Furthermore, the addition of *S. cerevisiae* made a significant contribution to the formation of alcohols and esters. Therefore, adding *L. plantarum* and *S. cerevisiae* can better improve the flavor and quality of fermented products. The results of sensory evaluation showed that Suanyu inoculated with *L. plantarum* and *S. cerevisiae* had stronger sensory characteristics, while the taste and odor properties were significantly improved. Overall, the combination of *L. plantarum* and *S. cerevisiae* could promote better proteolysis of Suanyu and the formation of non-volatile and volatile compounds. This research might offer several theoretical bases for the improvement of fermentation recipes and the industrialized manufacturing of excellent fermented goods.

## Figures and Tables

**Table 1:** Fermented Suanyu sensory score chart.

Index	Score			
	8–10	5–8	2–5	0–2
Overall acceptance	Like	Acceptable	General	Unacceptable
Texture	Strongly elastic	Elastic	Slightly elastic	inelastic
Gloss	Strongly glossy	Glossy	Slightly glossy	tarnish
Odor	Strong fermentation aroma	Fermentation aroma	Slight fermentation aroma	No fermentation aroma
Taste	Intense acid fragrance, intensely sweet and umami	Acid fragrance, slightly sweet and umami	Slight acid fragrance, slightly sweet and umami	No acid fragrance, no sweet or umami

**Table 2:** Changes of water content, TN, NPN, and PI values of Suanyu samples in each group during the whole fermentation process.

Time (Days)	TN (mg/g, d.w.)				NPN (mg/g, d.w.)				Protein Degradation Index (%)			
	NF	SC	LP	MF	NF	SC	LP	MF	NF	SC	LP	MF
0	65.03 ± 0.94 ^Aa^	65.03 ± 0.94 ^Aa^	65.03 ± 0.94 ^Aa^	65.03 ± 0.94 ^Aa^	2.35 ± 0.17 ^Ad^	2.35 ± 0.17 ^Ad^	2.35 ± 0.17 ^Ad^	2.35 ± 0.17 ^Ad^	3.61 ± 0.25 ^Ad^	3.61 ± 0.25 ^Ad^	3.61 ± 0.25 ^Ad^	3.61 ± 0.25 ^Ad^
5	68.93 ± 0.88 ^Aa^	68.65 ± 0.56 ^Aa^	68.10 ± 1.32 ^Aa^	67.81 ± 0.41 ^Aa^	4.24 ± 0.08 ^Dc^	4.60 ± 0.09 ^Cc^	5.28 ± 0.10 ^Bc^	5.86 ± 0.17 ^Ac^	6.15 ± 0.16 ^Dc^	6.70 ± 0.14 ^Cc^	7.76 ± 0.07 ^Bc^	8.65 ± 0.29 ^Ac^
10	67.68 ± 0.56 ^Aa^	69.62 ± 0.76 ^Aa^	69.20 ± 0.95 ^Aa^	68.50 ± 0.46 ^Aa^	6.25 ± 0.26 ^Db^	6.73 ± 0.05 ^Cb^	7.48 ± 0.25 ^Bb^	8.09 ± 0.15 ^Ab^	9.23 ± 0.30 ^Db^	9.67 ± 0.05 ^Cb^	10.82 ± 0.38 ^Bb^	11.81 ± 0.28 ^Ab^
15	70.18 ± 1.16 ^Aa^	70.01 ± 1.75 ^Aa^	69.97 ± 0.90 ^Aa^	69.29 ± 1.40 ^Aa^	7.56 ± 0.13 ^Da^	7.96 ± 0.05 ^Ca^	8.88 ± 0.14 ^Ba^	9.56 ± 0.10 ^Aa^	10.70 ± 0.30 ^Da^	11.38 ± 0.32 ^Ca^	12.69 ± 0.20 ^Ba^	13.79 ± 0.13 ^Aa^

Note: d.w. indicates dry weight. Reported values correspond to the mean ± standard deviation. Uppercase letters indicate significant differences between different treatment groups on the same day (*p* < 0.05); lowercase letters indicate significant differences within the same group across different fermentation times (*p* < 0.05).

## Data Availability

The original contributions presented in this study are included in the article/Supplementary Material. Further inquiries can be directed to the corresponding author.

## Supplementary Materials

The following supporting information can be downloaded at: https://www.mdpi.com/article/10.3390/foods15152626/s1, Table S1: Changes of FAA content in grass carp samples of each group during the whole fermentation process.
