# Supplementary material for: Effect of Lactiplantibacillus plantarum and Saccharomyces cerevisiae Inoculation on Quality of Suanyu: Focusing on the Protein Degradation and Flavor Profiles Analysis
Source: Foods. 2026 Jul 27;15(15):2626. doi: 10.3390/foods15152626 (PMC13465453; doi:10.3390/foods15152626)
Supplement: Supplementary file 1 [file foods-15-02626-s001.zip › foods-4410339-supplementary.pdf]

Supplementary S1

Table S1 Changes of FAA content in grass carp samples of each group during the whole fermentation process

Types	Taste characteristics	Threshold (mg/100 g)	Samples	Fermentation time (d)			
				0	5	10	15
Asparagine	umami (+)	100	NF	4.19±0.23^{Ad}	17.95±0.22^{Bc}	41.88±0.58^{Bb}	64.07±0.67^{Aa}
			SC	4.19±0.23^{Ad}	28.16±0.56^{Ac}	43.43±0.73^{Bb}	60.28±0.49^{Aa}
			LP	4.19±0.23^{Ad}	24.84±0.68^{Ac}	41.49±1.74^{Bb}	57.58±0.87^{Aa}
			MF	4.19±0.23^{Ad}	26.24±1.13^{Ac}	48.44±1.18^{Ab}	60.05±0.13^{Aa}
Threonine	sweet (+)	260	NF	7.26±0.31^{Ab}	2.38±0.16^{Bc}	8.43±0.75^{Bb}	12.15±0.45^{Ba}
			SC	7.26±0.31^{Ab}	6.71±0.11^{Bb}	12.93±0.19^{Ba}	13.09±0.16^{Ba}
			LP	7.26±0.31^{Aa}	5.24±0.16^{Bc}	3.65±0.13^{Bd}	6.77±0.21^{Ba}
			MF	7.26±0.31^{Ad}	33.78±0.66^{Ac}	71.37±0.37^{Ab}	101.80±1.65^{Aa}
Serine	sweet (+)	150	NF	6.86±0.22^{Ad}	12.57±0.46^{Ac}	18.42±1.09^{Bb}	20.65±0.85^{Ba}
			SC	6.86±0.22^{Ad}	13.61±0.41^{Ac}	25.09±0.77^{Ab}	28.32±0.65^{Aa}
			LP	6.86±0.22^{Ad}	10.63±0.55^{Bc}	14.86±0.51^{Cb}	16.21±0.71^{Ca}
			MF	6.86±0.22^{Ad}	13.03±0.43^{Ac}	19.25±0.83^{Bb}	21.04±0.78^{Ba}
Glutamic	umami (+)	30	NF	2.21±0.05^{Ad}	37.09±1.19^{Ac}	29.15±1.58^{Cb}	71.33±1.64^{Ba}
			SC	2.21±0.05^{Ad}	17.37±0.36^{Cc}	32.92±0.48^{Cb}	42.14±0.47^{Ca}
			LP	2.21±0.05^{Ad}	31.37±0.99^{Bc}	51.52±0.79^{Bb}	75.03±1.32^{Ba}
			MF	2.21±0.05^{Ad}	30.83±0.64^{Bc}	61.06±0.76^{Ab}	85.11±0.87^{Aa}
Glycine	sweet/umami (+)	130	NF	90.14±4.08^{Ab}	88.65±1.43^{Cb}	97.84±6.10^{Bab}	103.60±2.36^{Ba}
			SC	90.14±4.08^{Aa}	73.49±1.24^{Cc}	83.09±2.62^{Cb}	86.68±0.77^{Cab}
			LP	90.14±4.08^{Ab}	106.18±2.70^{Aa}	106.62±0.48^{Aa}	111.04±1.09^{Aa}
			MF	90.14±4.08^{Ab}	93.45±2.10^{Bb}	106.12±0.88^{Aa}	110.67±2.77^{Aa}
Alanine	sweet/umami (+)	60	NF	35.43±1.67^{Ac}	41.52±0.73^{Bc}	36.19±1.31^{Db}	72.69±1.86^{Ca}
			SC	35.43±1.67^{Ac}	37.18±0.16^{Cc}	41.58±0.94^{Cb}	46.66±0.52^{Da}
			LP	35.43±1.67^{Ad}	51.35±1.36^{Ac}	60.32±0.94^{Bb}	79.85±1.39^{Ba}
			MF	35.43±1.67^{Ad}	40.96±0.58^{Bc}	67.97±1.32^{Ab}	85.68±1.10^{Aa}
Cysteine	bitter/sweet/surfur (-)	ND	NF	6.52±2.59^{Ac}	4.89±0.19^{Cc}	40.07±1.75^{Bb}	106.19±2.77^{Aa}
			SC	6.52±2.59^{Ad}	19.91±0.81^{Bc}	35.38±0.44^{Bb}	52.96±0.75^{Ba}
			LP	6.52±2.59^{Ad}	45.41±0.28^{Ac}	69.91±1.96^{Ab}	96.65±0.21^{Aa}
			MF	6.52±2.59^{Aa}	3.21±0.63^{Cd}	3.67±0.15^{Cc}	4.60±0.17^{Cb}
Valine	sweet/bitter (-)	40	NF	4.50±0.31^{Ad}	6.07±0.04^{Ac}	6.68±0.18^{Ab}	7.18±0.05^{Aa}
			SC	4.50±0.31^{Ac}	4.00±0.23^{Bc}	4.62±0.08^{Bb}	5.62±0.20^{Ba}
			LP	4.50±0.31^{Aa}	2.38±0.11^{Cd}	3.13±0.13^{Cc}	3.84±0.10^{Cb}
			MF	4.50±0.31^{Aa}	2.29±0.12^{Cb}	2.68±0.20^{Db}	4.06±0.16^{Ca}
Methionine	bitter/sweet/surfur (-)	30	NF	0.04±0.01^{Ac}	0.45±0.03^{Cb}	0.44±0.05^{Cb}	1.22±0.08^{Ca}
			SC	0.04±0.01^{Ac}	0.67±0.17^{Ba}	0.30±0.06^{Cb}	0.29±0.03^{Db}
			LP	0.04±0.01^{Ad}	0.92±0.19^{Ac}	2.87±0.10^{Bb}	5.55±0.15^{Aa}
			MF	0.04±0.01^{Ac}	0.47±0.02^{Cc}	3.24±0.16^{Ab}	4.43±0.36^{Ba}
Isoleucine	bitter (-)	90	NF	9.66±0.03^{Ad}	34.13±0.65^{Ac}	25.19±0.83^{Bb}	79.05±1.95^{Ba}
			SC	9.66±0.03^{Ad}	13.09±0.22^{Cc}	19.55±0.15^{Bb}	26.05±0.55^{Ca}
			LP	9.66±0.03^{Ad}	37.24±0.38^{Ac}	66.00±0.63^{Ab}	94.41±0.63^{Aa}
			MF	9.66±0.03^{Ad}	26.86±0.44^{Bc}	67.23±1.40^{Ab}	96.05±1.18^{Aa}
Leucine	bitter (-)	190	NF	3.83±0.84^{Ac}	1.99±0.41^{Dc}	12.29±1.07^{Cb}	36.71±0.67^{Aa}
			SC	3.83±0.84^{Ad}	7.51±0.73^{Cc}	13.75±0.21^{Cb}	17.63±0.48^{Aa}
			LP	3.83±0.84^{Ad}	14.90±0.33^{Ac}	25.14±0.85^{Bb}	37.84±1.53^{Aa}
			MF	3.83±0.84^{Ad}	12.25±0.50^{Bc}	28.56±2.05^{Ab}	38.26±0.81^{Aa}
Tyrosine	bitter (-)	ND	NF	2.40±0.39^{Ad}	6.60±0.49^{Ac}	6.68±0.18^{Cb}	7.74±0.25^{Ba}
			SC	2.40±0.39b^{Ac}	1.99±0.05^{Bc}	2.60±0.08^{Db}	3.19±0.16^{Ca}
			LP	2.40±0.39^{Ac}	1.97±0..07^{Bc}	8.44±0.40^{Bb}	19.86±0.13^{Aa}
			MF	2.40±0.39^{Ad}	6.62±0.16^{Ac}	12.68±0.70^{Ab}	18.48±0.42^{Aa}
Phenylalanine	bitter (-)	90	NF	5.76±0.17^{Ad}	26.19±0.67^{Bc}	17.04±0.29^{Bb}	67.02±4.05^{Ba}
			SC	5.76±0.17^{Ad}	8.99±0.07^{Cc}	19.88±0.33^{Bb}	26.09±0.46^{Ca}
			LP	5.76±0.17^{Ad}	31.99±0.76^{Ac}	56.06±0.22^{Ab}	84.89±0.86^{Aa}
			MF	5.76±0.17^{Ad}	26.23±1.07^{Bc}	62.12±1.46^{Ab}	84.59±1.68^{Aa}
Lysine	sweet/bitter (-)	50	NF	17.16±0.19^{Ad}	42.54±0.78^{Ac}	27.32±1.97^{Cb}	49.73±0.90^{Ba}
			SC	17.16±0.19^{Ac}	18.15±0.17^{Dc}	27.35±0.68^{Cb}	34.83±0.36^{Ca}
			LP	17.16±0.19^{Ad}	35.59±0.52^{Bc}	50.48±0.87^{Bb}	80.20±1.14^{Aa}
			MF	17.16±0.19^{Ad}	31.80±0.87^{Cc}	60.63±1.20^{Ab}	79.70±1.49^{Aa}
Histidine	bitter (-)	20	NF	308.05±17.68^{Ac}	97.29±1.71^{Cb}	256.77±17.48^{Ca}	295.36±6.25^{Aa}

			SC	308.05±17.68^{Aa}	322.13±6.14^{Aa}	250.53±0.31^{Db}	202.70±2.40^{Cc}
			LP	308.05±17.68^{Aa}	279.32±3.65^{Bb}	262.79±5.68^{Bb}	238.24±2.61^{Bc}
			MF	308.05±17.68^{Aa}	265.74±7.97^{Bb}	238.39±6.32^{Dc}	232.71±4.26^{Bc}
Arginine	sweet/bitter（-)	50	NF	4.34±0.34^{Ab}	3.77±0.20^{Cb}	4.42±0.46^{Db}	10.61±0.28^{Ba}
			SC	4.34±0.34^{Ac}	3.99±0.09^{Cc}	8.15±0.23^{Cb}	9.10±0.09Ba
			LP	4.34±0.34^{Ad}	12.61±0.22^{Ac}	21.80±0.49^{Bb}	35.09±0.91^{Aa}
			MF	4.34±0.34^{Ad}	9.85±0.29^{Bc}	25.34±0.09^{Ab}	34.52±0.52^{Aa}
Proline	sweet/bitter（+)	300	NF	17.90±1.13^{Ac}	11.54±1.24^{Cb}	25.37±1.80^{Ba}	25.88±0.90^{Ba}
			SC	17.90±1.13^{Ab}	18.49±3.0^{Bb}	24.68±1.00^{Ba}	26.99±0.51^{Ba}
			LP	17.90±1.13^{Ac}	18.23±0.52^{Bb}	20.62±0.09^{Cb}	22.47±0.13^{Ba}
			MF	17.90±1.13^{Ad}	24.16±1.07^{Ac}	31.88±0.79^{Ab}	37.99±0.07^{Aa}

Note: (+) indicates pleasant taste; (-) indicates unpleasant taste; db indicates dry basis; ND indicates that the threshold was not detected. Reported values correspond to the mean ± standard deviation. Different letters in the same column indicate significant differences (P < 0.05).
